# Dementia registry in Guinea: clinical and etiological aspects at the Neurological Department of the Conakry teaching Hospital

**DOI:** 10.1002/alz70857_099458

**Published:** 2025-12-24

**Authors:** Guelngar Carlos Othon, Juste Milman Kadji, Foksouna Sakadi, Amara Cisse

**Affiliations:** ^1^ Department of Neurology, Ignace Deen National Hospital, Conakry, Conakry, Conakry, Guinea; ^2^ Department of Neurology, Reference Hospital, Ndjamena, NDdjamena, Ndjamena, Chad

## Abstract

**Background:**

In a tropical environment, etiological confirmation of dementia is difficult to establish due to under‐medicalization and delays in consultation. We carried out a retrospective study of 203 patients hospitalized for dementia syndrome for a period of 5 years in the Neurology department of the Conakry University Hospital Center with the aim of a clinical and etiological re‐evaluation.

**Method:**

This was a retrospective study of a descriptive type carried out between 2019 and 2024 based on the register of patients hospitalized for dementia syndrome meeting DSM‐IV criteria focused on highlighting disorders of higher functions combining both memory impairment and at least one of the four signs among language disorders, apraxia, agnosia and executive dysfunction. Thus the diagnostic criteria were based on the confirmation of higher function disorders by clinical tests: Mini Mental State Examination (MMSE) if possible. For patients with no educational level we used the 5‐word test. All received brain MRI imaging and a biological assessment.

**Result:**

The study included 203 cases of dementia confirmed according to DSM‐IV criteria, out of a total of 1049 patients, i.e. a hospital frequency of 19.35%. Etiologically, the patients were classified as follows: 43 cases of vascular dementia 38 cases of syphilitic dementia, 33 cases of Alzheimer's type, 29 cases of toxic dementia (aluminum), 20 cases of Huntington's disease, dementia with Lewy bodies 12 cases, 10 cases of fronto‐temporal type dementia, normal pressure hydrocephalus in 7 cases, Gayet Wernicke encephalopathy in 3 cases, dementia AIDS in 5 cases, and 3 cases of Parkinson's disease.

**Conclusion:**

This study shows a clinical and etiological profile of the spectrum of dementia in a tropical environment in a context of under‐medicalization.

